# Photonic resonator absorption microscopy: why consider metallic and magneto-plasmonic nano-assemblies over bare nanoparticles for digital biosensing?

**DOI:** 10.1007/s00216-025-06056-y

**Published:** 2025-08-30

**Authors:** Skye Shepherd, Weinan Liu, Seemesh Bhaskar, Brian T. Cunningham

**Affiliations:** 1 Nick Holonyak Jr. Micro and Nanotechnology Laboratory, University of Illinois at Urbana-Champaign, Urbana, IL 61801 USA; 2 Department of Electrical and Computer Engineering, University of Illinois at Urbana-Champaign, Urbana, IL 61801 USA; 3 Department of Bioengineering, University of Illinois at Urbana-Champaign, Urbana, IL 61801 USA; 4 Carl R. Woese Institute for Genomic Biology, University of Illinois at Urbana-Champaign, Urbana, IL 61801 USA; 5 Department of Chemistry, University of Illinois at Urbana-Champaign, Urbana, IL 61801 USA; 6 Cancer Center at Illinois, Urbana, IL 61801 USA

**Keywords:** Photonic crystal, Enhanced absorption, Magnetic hotspot, Cryosoret, Guided-mode resonance, COMSOL

## Abstract

The unique optical interaction of species such as nanomaterials, proteins, viruses, antibodies, microRNA, and exosomes with the one-dimensional grating-based photonic crystals (PCs) has been leveraged in their detection using photonic crystal absorption microscopy (PRAM). While the principle and fundamental mechanism of such interfacial interactions are well delineated using wavelength and intensity modulations associated with the guided-mode resonance (GMR) of the PC, the effect of nano-assemblies in place of nanoparticles (NPs) has not been reported previously. In this work, the fundamental limitations observed with pristine NPs are overcome through the use of tunable AuNP assemblies synthesized via adiabatic cooling technology, where tunable nano-assemblies are obtained by subjecting the respective NPs to − 196 °C. Moreover, the higher contrast rendered by magneto-plasmonic, Fe_3_O_4_-Au hybrid nano-assemblies vis-à-vis metallic AuNP assemblies is corroborated with COMSOL Multiphysics simulations using electric and magnetic field hotspots. The high-contrast digital resolution enabled by magneto-plasmonic hybrid nano-assemblies, on account of synergistic coupling between the GMR of the underlying PC, delocalized Bragg, and localized Mie plasmons of dielectric-metal nano-assemblies, demonstrated excellent performance for microRNA-375-3p detection, opening a new window to explore hybrids of tunable “permittivity + permeability” as active probes in the design and development of microscopy-based biosensing modalities.

**Graphical Abstract:**

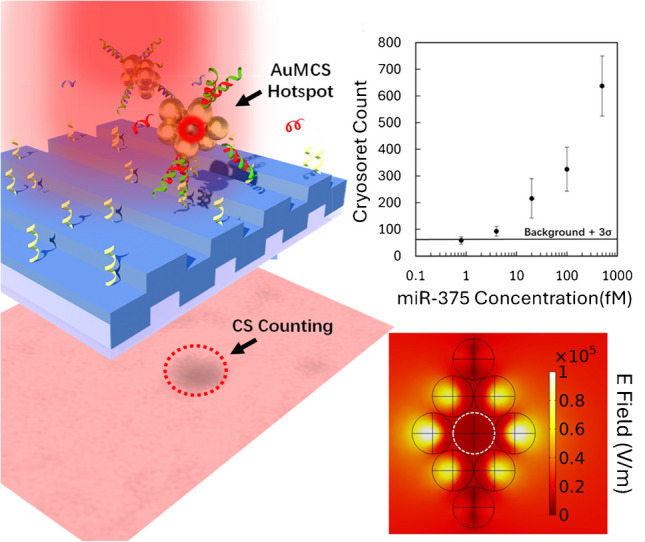

## Introduction

Photonic resonator absorption microscopy (PRAM) has emerged as a transformative approach in label-free biosensing, particularly on account of its ability to provide “digital” resolution of individual target molecules with a high signal-to-noise ratio [1–3]. PRAM utilizes photonic crystals (PCs) as a biosensing platform, leveraging their unique optical properties to achieve high-contrast imaging and counting, without complex enzymatic amplification and preincubation, which is highly desirable for point-of-care diagnostics [4, 5]. The significance of label-free imaging stems from its ability to detect biomolecular interactions without the need for fluorescent or radioactive labels, offering a more direct and physiologically relevant assessment of biomolecular interactions, including but not limited to molecule-protein, protein–protein, and cell-drug interactions [4]. PCs of various types are well-explored for biosensing applications due to their capability to support several resonances such as Bloch surface wave [6], Fano resonances [7], internal optical modes [8], Fabry–Perot resonances [9], bound states in the continuum (BICs) [10], and guided-mode resonance (GMR) [11, 12], which enhances light-matter interactions and enables highly sensitive detection [13, 14]. Augmented absorption of light at the interface of two systems in a micro-nano-environment has been the topic of interest in areas such as diffraction gratings [15, 16], coherent absorption [17], band nesting [18], and metamaterials [19, 20]. In the past few decades, our group has demonstrated the practical applicability of imaging-based digital counting methods using the GMR effects and enhanced absorption, scattering, and interference of light at the PC interface for detecting individual biomolecular interactions by mapping resonance shifts within a field of view, leading to applications in cancer diagnostics [21–25]. Particularly, PRAM technology has been utilized for the detection of DNA, circulating exosomal microRNA (miRNA), SARS-CoV-2 antigen, specific antibodies, proteins, and enzymes [1, 2, 26–29].

Fundamentally, the operating principle of PRAM relies on the ability of a PC to act as an optical transducer consisting of a periodic arrangement of low and high refractive index materials. When illuminated with broadband light, diffraction causes selective coupling of light into the high-index layer, leading to constructive and destructive interference effects. At a specific resonance condition, nearly all incident light is reflected, producing a sharp optical signal highly sensitive to surface perturbations [30, 31]. The presence of biomolecules or bio-analyte tagged plasmonic nanoparticles (NPs) alters the local refractive index (RI), modifying the peak wavelength value (PWV) and intensity of the reflected light. By mapping these changes, PRAM provides a direct visualization of surface-molecule interactions, cell adhesion, and nanoparticle binding, offering a quantitative and qualitative means for biosensing [4, 30–32]. Despite these advancements, signal contrast rendered by PRAM instrument is limited due to dissimilarities in resonance interactions, particularly when utilizing plasmonic nanomaterials of sharp morphologies as contrast agents [33]. This limitation emerges on account of the intrinsic properties of the plasmonic nanomaterials as their localized surface plasmon resonance (LSPR) is extremely sensitive to the structural morphology (shape), size, composition, as well as the dielectric constant of the local micro-nano-environment [34, 35]. For instance, nano-urchins that are tagged to bio-analytes function by interacting with the guided-mode resonance of the PC, modulating the resonant wavelength based on changes in local refractive index. However, these nano-urchins that are tuned to match the PC resonance exhibit shifts in LSPR due to deviations in nano-spike morphology [36, 37]. That is, their performance is influenced by variations in their surface topology, as even slight morphological deviations can lead to unintended red or blue shifts in resonance, thereby diminishing detection precision [38–41]. Additionally, we have developed core–shell magneto-plasmonic nano-urchins, which improve detection speed and sensitivity by incorporating magneto-plasmonic effects [2]. While these structures have shown promising results in accelerating assay dynamics, the magnetic core remains embedded within the plasmonic shell, limiting the full exploitation of “magneticplasmons-PC resonance-EM field” interactions at the biosensor interface. Moreover, surface interactions with analytes sometimes compromise the sharp curvature of nano-urchins, leading to blunting effects that further alter their plasmonic sensing response.

Recently, engineering plasmonic nano-assemblies has emerged with creative solutions to the limitations posed by pristine plasmonic nanomaterials in the domain of imaging and spectroscopic applications [42, 43]. The near-field coupling between the neighboring plasmonic nanomaterials demonstrates nano-gap-based antenna effect which has electric field enhancement effects much greater than those of individual nano-constructs [44–47]. In this context, dimeric, trimeric, tetrameric, octameric, and similarly multimeric core-satellite structures, and nanochains sustaining higher orders of coupled modes have been investigated showcasing interesting concepts from a physics principles perspective as well as applications in dark-field imaging, fluorescence imaging, SPR imaging, and surface-enhanced Raman scattering (SERS) imaging to name a few [43, 44, 47–51]. Among the evolving numerous techniques for the generation of oriented, templated, and directed nano-assembly, template-less, linker-less, and surfactant-free methods based on physical stimuli such as electric field, magnetic field, light-driven, and temperature-dependent approaches have emerged with several advantages as compared to those that are dependent on chemical stimuli (such as pH, ions, acid/base, molecule-triggered nano-assembly) [46, 47]. Among such temperature-stimuli-based self-assembly routes, the cryosoret nano-engineering (CSNE) methodology provides superior optical and plasmonic properties due to collective and coherent resonance effects rendering “hottest hotspots” [52, 53]. Specifically, cryosorets exhibit delocalized Bragg and localized Mie plasmons, making them particularly effective for biosensing applications with applications in single molecule detection and interferometric scattering microscopy [53, 54]. While interfaced with the PC substrate, the synergistic interplay between resonance modes of PC and cryosorets enhances the signal contrast and its stability, reducing the impact of morphological variations seen in isolated nanoparticles [55]. Additionally, their application in sensing a diverse array of analytes, ranging from ions to small molecules, underscores their versatility and efficacy in high-sensitivity detection platforms.

Moreover, while plasmonic nanomaterials have revolutionized the manipulation of light at the nanoscale, their optical response has predominantly relied on electric field (E-field) interactions due to the absence of intrinsic magnetic moments [56–58]. As such, traditional plasmonic structures, including noble metal nanoparticles, are limited in sustaining magnetic field (H-field) enhancements, particularly within their bulk. To overcome this constraint, recent advances have turned toward high refractive index (HRI) dielectric nanomaterials, which exhibit strong Mie resonances and can simultaneously support E-field and H-field confinement [58–64]. Materials such as Fe₃O₄ offer additional advantages by not only being HRI dielectrics but also exhibiting intrinsic magnetic flux densities. By integrating plasmonic and dielectric components within engineered nano-assemblies, it becomes possible to generate hybrid electromagnetic hotspots—regions of co-localized E-field and H-field enhancement—particularly in nanoscale gaps [2, 65, 66]. These magnetic hotspots introduce a new degree of control over light-matter interactions and can significantly amplify photonic processes such as fluorescence [67, 68]. Motivated by these insights, this study demonstrates a hybrid architecture that synergistically combines Au nanoparticles and Fe₃O₄ nanoparticles within a photonic crystal platform. This approach leverages the dual-field enhancement capabilities to surpass the limitations of previous nano-assemblies, enabling improved signal intensities and spectral control in advanced optical sensing applications.

In this background here, we introduce a novel strategy by synthesizing cryosoret nano-assemblies composed of plasmonic gold (Au) and magnetic iron oxide (Fe_3_O_4_) nanoparticles, leveraging their combined potential to generate electric and magnetic hotspots. The integration of both materials allows us to exploit unique permittivity-permeability interactions, leading to enhanced optical and EM responses, unlike the conventional hotspots that are only enhancements in the electric field intensity (as in individual metal NPs) [57, 59, 69]. The circulating current densities [70, 71] in these hybrid nano-assemblies generated by three-dimensional nano-gaps further augment contributions from the permeability component of the magnetic Fe_3_O_4_ NPs [65, 66, 72]. Moreover, by incorporating Fe_3_O_4_ NPs, the nano-assemblies gain additional unique functionality in the form of tunable magnetic properties, which can be externally manipulated to optimize contrast and signal resolution. This approach not only improves the signal-to-noise ratio in PRAM but also introduces a new paradigm in nano-assembly design for imaging-based sensing applications. The interplay between localized Mie and delocalized Bragg resonances within the plasmonic-magnetic nano-assemblies [55] presents a compelling avenue for advancing high-contrast imaging with the proof-of-principle for absorption-based precision imaging technology presented in this work.

The hybridization of PRAM with magneto-plasmonic cryosoret nano-assemblies presents a quintessential convergence of PC technology and magneto-plasmonic nano-assemblies at the micro-nano-interface. While our experimental PRAM images present significantly higher contrast for cryosorets vis-à-vis individual nanoparticles, the trend observed for increasing numbers of nanoparticles per assembly is scrutinized both experimentally and using computer-aided COMSOL Multiphysics simulations. Although previous studies have demonstrated the benefits of integrating PCs with nano-assemblies for enhanced sensing applications (spectroscopic and microscopic) [53, 55, 73], our approach uniquely exploits the dual plasmonic and magnetic nature of the hybrid nano-constructs, for the first time to the best of our knowledge. The combination of GMR from PCs with the tunable plasmonic-magnetic interactions of cryosoret nano-assemblies introduces a new approach for digital biosensing that enhances the performance of PRAM beyond conventional limitations mentioned above. This integration enables higher signal contrast, improved analyte-magnetic NP interaction, and greater robustness against environmental fluctuations. Furthermore, the ability to modulate resonance conditions via external magnetic fields offers unprecedented control over biosensing dynamics, paving the way for next-generation label-free imaging platforms. By such interfacial engineering methods, we aim to establish a new standard in photo-plasmonic biosensing, exploiting the fundamental physics of PCs and magneto-plasmonic interactions, using simulation models and experimental methods, to advance the biosensing modalities.

## Experiment and simulation

The photonic crystal enhanced absorption (PRAM) is a biosensor microscopy platform developed by our team, with details of principle and mechanism of operation presented in earlier works [5, 26, 74]. Briefly, the basic components of the setup include the LED with a range from about 620 to 640 nm that is illuminated onto the PC substrate (from the non-patterned side), using collimating lenses and a polarizer (Fig. 5a). The polarized light is used to excite the specific polarization selective resonance of the PC (transverse magnetic in our case). Typically, in principle, the PC substrate reflects only a narrow band of the LED wavelengths with approximately 100% reflection efficiency into an uncooled silicon CMOS camera [5, 26]. In the presence of the plasmonic nanomaterial interfaced on the patterned PC substrate, based on the magnitude of absorption rendered by the plasmonic nanomaterial, the reflection intensity changes. Based on this primary concept, our lab has demonstrated the detection of different analytes by using capture ssDNA, toehold—probe on a NP and target analyte such as microRNA, where only in the presence of microRNA the nanoparticle is captured on the PC substrate and individually counted—hence the name “digital detection” [1, 2, 4, 27, 75]. However, this method highly relies on the ability of the nanomaterial to actively function as light absorbing species at the resonance wavelength of the PC. Since the absorption coefficient gets modified (typically hampered) by functionalization with larger sized biomolecules, there is a lookout for alternative approaches to overcome this limitation.

Here, we present the recently developed cryosoret nano-engineering method for achieving this objective. The details of cryosoret engineering method are discussed in our recent works [53, 62, 73, 76]. In brief, the nanoparticles are subjected to − 196 °C by immersing in LN2 for different time intervals. The thermo-migration induced by the cooling effect results in the generation of nano-assemblies where the adiabatic cooling is overcome by electrostatic repulsion. The synthesized NPs and nano-assemblies were characterized using Asylum Research MFP-3D atomic force microscopy (AFM), Hitachi S-4800 high-resolution scanning electron microscopy (SEM), Au–Pd Sputter Coater—Emscope SC 500, and JEOL 2100 CRYO transmission electron microscopy (TEM).

Thus, obtained NPs and nano-assemblies are later drop-casted over the clean PC substrate and observed under the PRAM optical setup in line with our earlier work [55]. The PC substrate was cleaned using isopropanol, acetone, and deionized water by sonicating for 3 min each, followed by drying using N2. The drop-casted nanomaterials (nanoparticles and cryosoret nano-assemblies) were incubated on top of the PC substrate in molecular grade water for 1 h at room temperature before imaging using the PRAM instrument [4, 74, 77]. The TEM images of multiple cryosorets (nano-assemblies obtained via cooling nanoparticles to cryo-environment) are used to develop nano-constructs for simulating their optical response in COMSOL Multiphysics software [76].

### Sequences

Purchased from Integrated DNA Technologies (Coralville, IA).

Probe-375: CCC ACC TAC ATC ACG CGA GCC GAA CGA ACT TTT TTT TTT TTT TT/3ThioMC3-D/.

Protector-375: GTT CGG CTC GCG TGA TGT AGG.

Capture: TGT AGG TGG GTT TTT TTT TTT TTT TTT TTT/3AmMO/.

Target (miR-375): rUrUrUrGrUrUrCrGrUrUrCrGr GrCrUrCrG rCrGrUrGrA.

### Probe DNA reduction and protector annealing

The thiolated probe was reduced using Tris(2-carboxyethyl)phosphine (TCEP) hydrochloride purchased from Sigma-Aldrich (C4706-2G). Four hundred fifty microliters of 100 µM of probe DNA was added to 50 µL of 50 mM TCEP solution and reduced for 2 h at room temperature. The excess TCEP and byproducts were removed by using 0.5 mL 3 kDa MW Amicon Ultra Centrifugal Filters (Millipore, UFC500308) by centrifuging for 15 min at 14.0 k rcf twice, then washing with 400 µL of molecular grade water. The reduced and concentrated probe was recovered by flipping the filter and centrifuging at 3.0 k rcf for 4 min. The final probe concentration was tested using a NanoDrop One (ThermoScientific), then was diluted in 1 × TE to 100 µM. The protector and thiolated probe sequences were then annealed at a 1:1.5 ratio with the protector in excess in a 1xTE, 12.5 mM MgCl_2_ solution by heating to 85 °C then cooling to room temperature. The reduced probe-protector duplex was used immediately for nanoparticle conjugation or was stored at − 20 °C for later use.

### Nanoparticle conjugation and cryosoret formation

Twenty milliliters of 20-nm-diameter small gold nanoparticles was incubated with 100 µL of 100 µM of the reduced thiolated probe-protector duplex for 48 h at room temperature in 1 × TE, 5 mM MgCl_2_. The AuNPs were then centrifuged three times at 10 k rcf for 30 min to remove unattached probe-protector, and were resuspended in 10 mL 1 × TE, 5 mM MgCl_2_ for cryosoret formation. Cryosorets were then formed using the pre-functionalized AuNPs and 20-nm magnetic nanoparticles for AuMCS4 (as this variant yielded the highest contrast in the PRAM studies). DNA-functionalized cryosoret assemblies were stored for up to 3 weeks at 4 °C.

### Surface functionalization of PCs and DNA capture attachment

Photonic crystal chips (1.2 by 1 cm) were glued to coverslips using UV-curable glue (Norland Optical Adhesives 63). The PCs were washed by sonicating for 2 min in acetone, then isopropanol, and then MilliQ® water. PCs were dried using nitrogen gas and heated to 80 °C for 20 min to dry completely. The PCs were oxygen plasma-treated using a PicoDiener machine at 100% power and 0.1 mbar, then were added to a 2% APTES solution in tetrahydrofuran (THF) for 1 h and shaken at 400 rpm at room temperature. Excess unbound silane was removed by sonicating for 2 min each in THF, acetone, and MilliQ® water. Thin cured polydimethylsiloxane (PDMS) was cut to create six 20-µL wells, attached to the PC surface using pressure to create separate sample wells. A solution of 100 mM N,N′-disuccinimidyl carbonate (Sigma-Aldrich) in 10% dimethyl sulfoxide (DMSO) was added for 30 min at room temperature, and then excess DSC was removed using three washes of 1% DMSO. The amino-functionalized capture DNA sequence was then reacted with a 50-µM concentration of capture in water with the DSC-functionalized surface for 3 h at room temperature. Excess capture was removed through washing the surface five times with 1 × TE, Tween20. Finally, the PC surface was blocked for 30 min using PBS SuperBlock (Invitrogen, #37,515). PCs were then immediately used for the PRAM-cryosoret assay.

### Detection of miR375 using PRAM-cryosoret assay

Reagents were added into a 20-µL reaction volume. The assay tested varying concentrations of miR-375 in a five-fold serial dilution (0 fM, 0.8 fM, 4 fM, 20 fM, 100 fM, and 500 fM). The probe-protector cryosorets were added into the well at a final concentration of 1 OD, in 1 × TE, 5 mM MgCl_2_, along with the target miR-375. These were allowed to react for 30 min before imaging. Three images were taken per well for each target concentration, and the assay for each concentration was repeated in triplicate. Images were processed using code previously detailed in [54, 73, 74] with a maximally stable external regions (MSER)–based counting method.

## Results and discussion

**Fig. 1:**
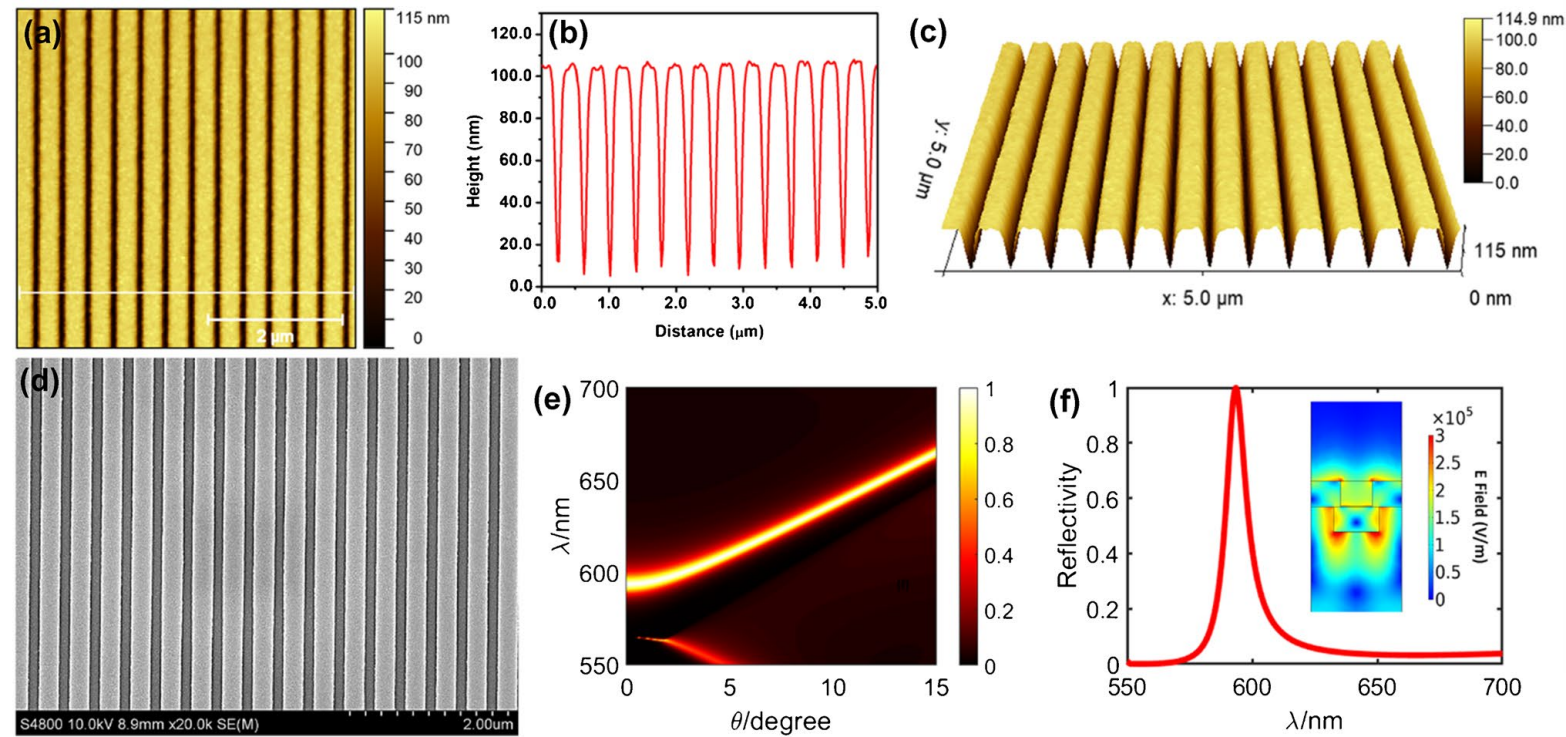
Structural, morphological, and field distribution of photonic crystal substrate. **a** AFM image showing white light along which the height information is extracted and plotted in **b**. **c** Three-dimensional topographical image of the AFM data shown in **a**. **c** SEM image showing the top view of the PC substrate. **e** Simulated wavelength vs. angle dispersion diagram of the PC substrate. **f** Simulated reflectance data of the PC substrate obtained at 0° for TM polarization shown with the inset capturing the hotspot E-field intensity distribution

We start by presenting the comprehensive characterization of the substrate and nanomaterials used for the experimentation. The substrate is a grating-based one-dimensional photonic crystal (PC) that has a sub-wavelength nano-structure with a periodic arrangement of a low refractive index material (silica) coated with a high refractive index (HRI) material (titania). The atomic force microscopy (AFM) image of the PC under investigation is presented in Fig. 1a, with a height profile along the white line shown in Fig. 1b. This, along with the three-dimensional view of the PC shown in Fig. 1c, presents topographical information of the grating interface (with the depth of the grooves being ~ 90 nm). Moreover, the low-resolution scanning electron microscopy (SEM) image shown in Fig. 1d presents the top view confirming the high uniformity of the grating morphology.

The dispersion diagram of the optically active substrate such as metallic thin films, metal-dielectric-metal (MDM) interfaces, and PC presents the transmittance or reflectance response at various wavelengths and angles [78–80]. The dispersion diagram for the PC in this work is shown in Fig. 1e, presenting characteristic guided-mode resonance (GMR) modes that arise due to the presence of guiding layer and diffraction elements [81, 82]. The diffracted and guided modes generated upon illumination of white light result in coupling of light into and out of the HRI material, as a consequence of which the destructive interference occurs at zeroth-order transmitted light, resulting in nearly 100% reflection efficiency [11, 83]. The optical response of such PC for all the angles from 0 to 30° under white light illumination is presented in our recent work [62]. It is under this specific wavelength of resonance (GMR) the evanescent field is generated on the top surface of the grating, extending to ~ 100 nm out of the grating PC substrate. The simulated transmittance dip is shown in Fig. 1f, with the inset presenting the E-field intensity distribution, showing the hotspots at the sharp tips of the grating PC (electric field standing waves).

Interfacing a nano-object at this grating interface at the excitation of GMR of the PC results in optical interaction of such nano-object with the evanescent field generated at resonance. This optical interaction can alter the original intrinsic scattering and absorption effects of the PC and nano-object, which results in either enhanced/quenched scattering and/or absorption. While the photonic crystal interferometric scattering microscopy (PRISM) technology developed by our group heavily relies on the scattering efficiency of the nano-objects [84–86], the photonic resonator absorption microscopy (PRAM) is based on the absorption efficiency of nano-objects, where the interactions render an image in the CCD camera due to resonant optical coupling [1, 2, 87]. Although our earlier works demonstrated substantial changes in near-field distributions, resonant wavelength maximum, and the intensity of the resonant peak (transmittance dip) for interfacing different types of nanomaterials including metallic (AuNPs) and dielectric (TiO_2_) [4], all such studies have been limited to a specific size of the nanomaterial. Variations in the size would shift the resonant absorption peak of NPs, thereby resulting in hampered coupling effects. To overcome such drawbacks, we demonstrate the utility of nano-assembly made up of pristine plasmonic gold cryosoret (CS) nano-assemblies and hybrid gold-magnetic cryosorets (MCS) nano-assemblies.

**Fig. 2:**
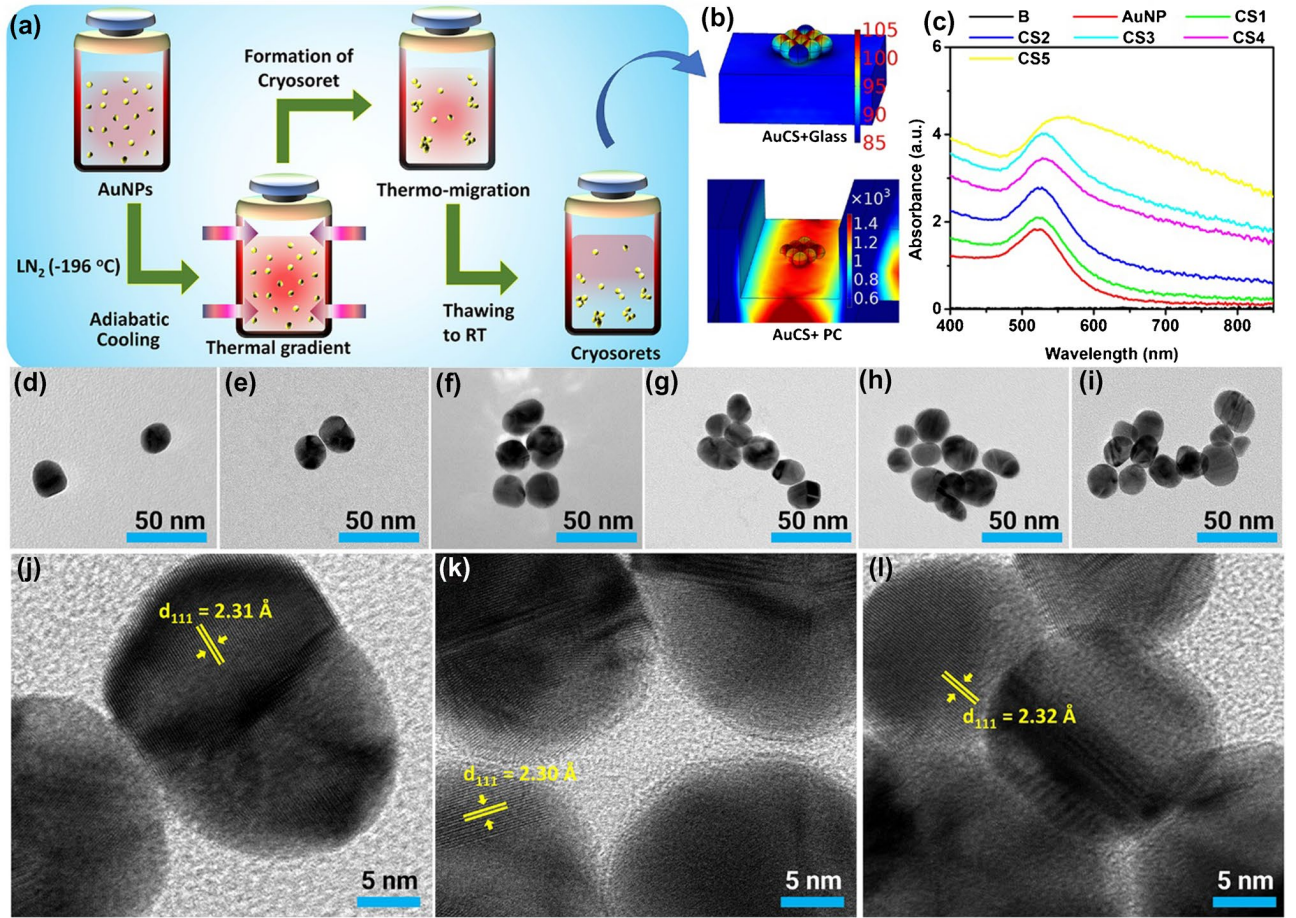
Synthesis, optical, and electron microscopy characterization of gold cryosorets. **a** Cryosoret nano-engineering method showcasing the steps involved in the generation of AuCSs. **b** Representative simulation of AuCS on glass and PC substrate. **c** Absorbance spectra of all the synthesized gold CSs. **d** TEM image of pristine AuNPs. **e**–**i** TEM images of gold CSs [1-5] where the numbers 1–5 represent the samples cooled at 15 s, 30 s, 1 min, 2 min, and 3 min, respectively. High-resolution TEM (HRTEM) images are shown in **j**, **k**, and **l** corresponding to **e**, **f**, and **g**, respectively

The crysoret nano-engineering methodology is presented in Fig. 2a conceptually, where the homogenous solution of plasmonic AuNPs subjected to adiabatic cooling results in the generation of gold cryosoret nano-assemblies. A representative 3D simulation of AuCS on glass and PC shown in Fig. 2b clearly indicates the increase in field intensity observed for the AuCS-PC interface. The absorbance spectra of pristine AuNPs show a characteristic LSPR at ~ 530 nm. The CSs 1–5 are obtained by cooling the AuNP solution to − 196 °C at 15 s, 30 s, 1 min, 2 min, and 3 min, respectively [53, 62, 76]. We observe a gradual broadening of the transverse LSPR mode and simultaneous occurrence of longitudinal modes at higher wavelengths, which are attributed to the generation of anisotropic chain-like nano-assemblies with delocalized Bragg plasmons, in accordance with earlier works [53–55]. Further, the TEM image of pristine AuNPs is shown in Fig. 2d, and Fig. 2e–i presents the TEM images of CSs of gold, presenting an increasing number of AuNPs per assembly. While representative images are presented here, multiple TEM images for each variant are extensively characterized and reported in earlier works [53, 62, 76]. The HRTEM images of representative cryosorets presented in Fig. 2j, k, l show the occurrence of multiple nano-gaps in and around the cryosoret nano-assembly. Such nano-gaps generate gap-induced Bragg scattering modes that results in the observation of spectral broadening as well as longitudinal modes. In this work, we have specifically chosen the AuNPs that show absorbance maximum at values far lower than that of the GMR of the PC to confirm our understanding of resonance coupling. Basically, we aim to validate that even if the nanomaterial does not have LSPR mode at PC resonance, assembling them would yield nanostructures that would be beneficial for digital resolution-based PRAM. That is, if larger size AuNPs are chosen, then there would be non-negligible absorption at the resonance of PC (~ 630 nm), hence increasing the probability of absorption-induced quenching of PC resonance. The AuNPs chosen here, of size ~ 20 nm, yield negligible absorption at the PC resonance wavelength, hence serving as an ideal candidate to verify our original hypothesis.

Harnessing the full potential of nanophotonics and magnetism requires careful convergence of associated complementary material platforms. Plasmonic and magnetic nanostructures have each carved distinct but influential trajectories in optical sensing, imaging, and modulation [65, 72, 88]. Yet, it is the hybridization of these two domains—plasmonics and magnetism—that offers a transformative leap, revealing synergistic functionalities that neither system could achieve by themselves. Applications pertaining to plasmonic NPs emerge on account of their ability to support LSPR, which is collective oscillations of conduction electrons that confine light into sub-wavelength regimes [89, 90]. This effect generates intense local electric fields, enabling dramatic enhancements in optical absorption, fluorescence, and Raman scattering. As such, they have been central to the evolution of ultrasensitive biosensors, photothermal therapies, and optical antennas. On the other hand, magnetic nanoparticles (MNPs), like Fe₃O₄, provide a dynamic modality: the ability to respond to external magnetic fields for actuation, spatial control, or signal modulation. Moreover, magnetic nanostructures exhibit magneto-optical (MO) effects such as the Faraday and Kerr effects, which arise due to their non-zero off-diagonal dielectric permittivity components, and such effects have been central to technologies involving non-reciprocal light propagation, magnetic field sensing, and active optical switching [65, 72, 88].

It is important to note that despite their individual merits, each system suffers from intrinsic limitations. Magnetic nanomaterials tend to be optically lossy due to suppressed plasmonic resonances. Conversely, while noble metals support strong plasmonic effects, they exhibit weak magneto-optical responses [91, 92]. This dichotomy has spurred intense research into the synthesis and application of magneto-plasmonic nano-constructs, wherein magnetic and plasmonic components are either spatially juxtaposed (as multi-layers for instance) or co-integrated into single hybrid nano-constructs. Such combinations allow external magnetic fields to modulate plasmonic behavior and vice versa, paving the way for tunable sensors, direction-sensitive optics, and enhanced MO signals through field confinement.

**Fig. 3:**
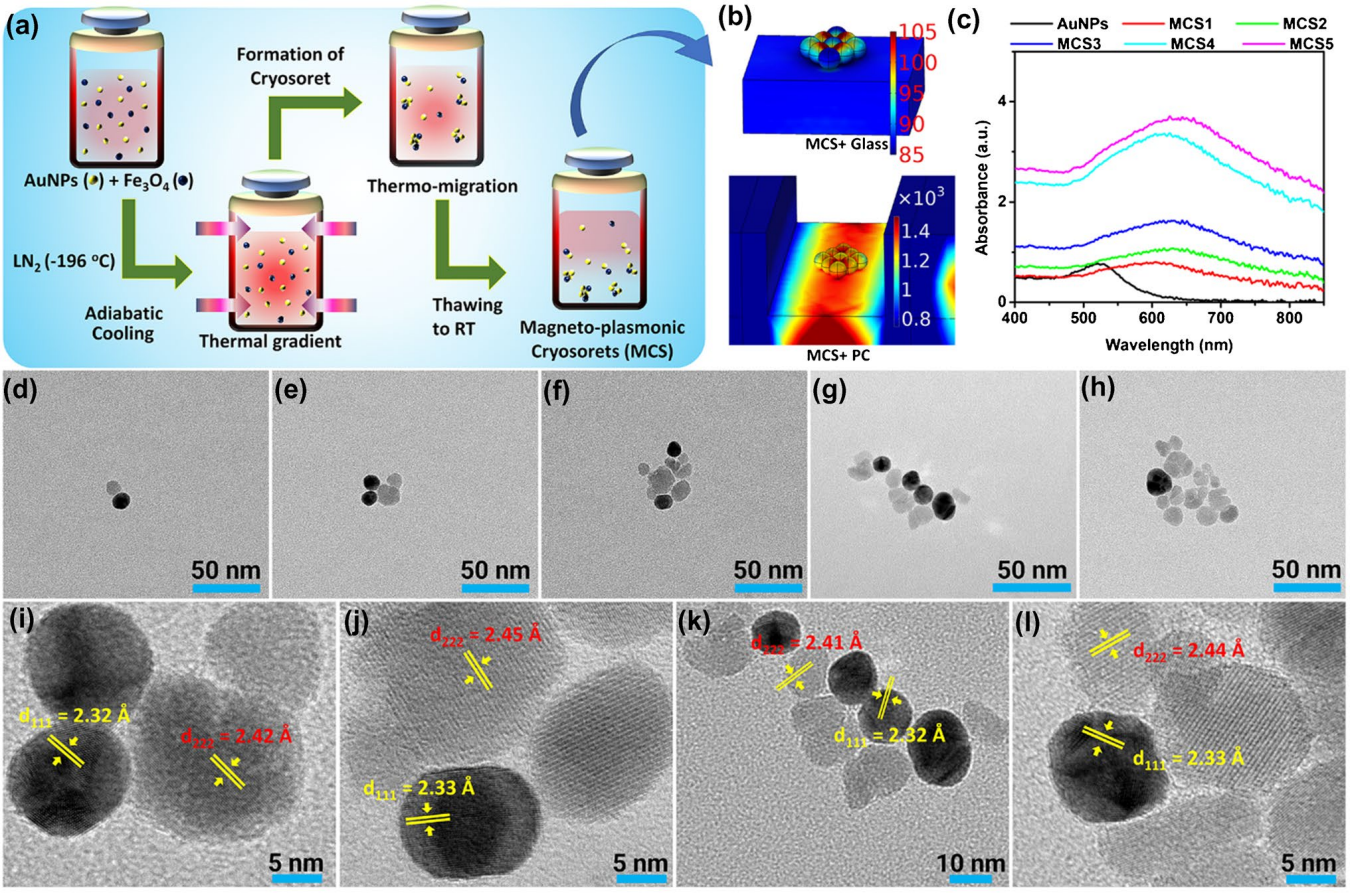
Synthesis, optical, and electron microscopy characterization of magneto-plasmonic hybrid cryosorets. **a** Cryosoret nano-engineering method showcasing the steps involved in the generation of MCSs. **b** Representative simulation of MCS over glass and PC substrate. **c** Absorbance spectra of all the synthesized MCSs. **d**–**h** TEM images of MCSs [1-5] where the numbers 1–5 represent the samples cooled at 15 s, 30 s, 1 min, 2 min, and 3 min, respectively. High-resolution TEM (HRTEM) images are shown in **i**, **j**, **k**, and **l** corresponding to **e**, **f**, **g**, and **h**, respectively

In this background, we present a new class of linker-less hybrid nano-assemblies composed of AuNPs and Fe₃O₄ magnetic NPs with the synthesis method shown in Fig. 3a. A homogenous mixture of AuNPs and Fe₃O₄ NPs was mixed in a 1:1 ratio and subjected to the adiabatic cooling method [52, 54, 62]. The enhanced field coupling between the magneto-plasmonic cryosorets (MCSs) and the PC, as compared to glass, is shown for a representative case in Fig. 3b. Absorbance spectra of all the synthesized MCSs [1-5] are presented in Fig. 3c, where a red-shift in the LSPR of the pristine AuNPs is observed with broadening of the spectra observed for an increasing number of NPs per assembly. Furthermore, the TEM images of the MCS [1-5] are shown in Fig. 3d–h, clearly indicating the anisotropic arrangement of plasmonic AuNPs and Fe₃O₄ NPs in the formation of hybrid cryosorets presenting three-dimensionally distributed hotspots. Also, a few representative HRTEM images are presented along with the lattice fringes characterizing the plasmonic Au (Inorganic Crystal Structure Database, ICSD reference code: 98–061-1625) and dielectric Fe₃O₄ NPs (ICSD reference code: 98–008-5807). The high metallic and dielectric nature of the Au and Fe₃O₄ comprising the nano-assemblies are vividly seen due to high and marginal scattering of the electron beam in TEM measurements, rendering the former with dark black and the latter with a light gray color. These well-characterized nanomaterials are further used to build representative models in COMSOL Multiphysics software to comprehend the effect of such multi-particle arrangement in modulating the local E- and H-field intensities.

The study of EM field interactions at the micro-nanoscale remains a cornerstone in advancing plasmonic and magneto-plasmonic explorations. Although the LSPR in metal NPs concentrates electric fields (E-fields) into sub-wavelength volumes, creating E-field hotspots that dramatically enhance light-matter interaction, the magnetic component of light (H-field) is typically weak in conventional plasmonic systems due to the low magnetic permeability at optical frequencies [59, 88, 93]. Recent studies in magneto-plasmonic nanoarchitectures have opened new avenues to co-localize and amplify both E-field and H-field intensities, enabling applications in enhanced spectroscopy, active photonic devices, and biosensing platforms. In this perspective, several works have reported the tactical use of such hybrid assemblies to achieve tailored field distributions. Maksymov et al. reviewed the role of magneto-plasmonic nanoantennas, demonstrating ferromagnetic and plasmonic constituents enabling enhanced magneto-optical effects and dual-mode control of light via E-field and H-field hotspots [94]. Luong et al. investigated Ag-Co composite nanostructures and noted that Faraday rotation could be maximized at the LSPR wavelength, which is a direct consequence of intense E-field concentration and H-field enhancement coexisting in a composite structure [95]. Moreover, the ability of DNA-origami-based magnetic NP rings to simultaneously sustain E-field and H-field dipolar resonances producing tunable magnetic Fano resonances and SPPs in structured networks was reported by Wang et al. [64].

**Fig. 4:**
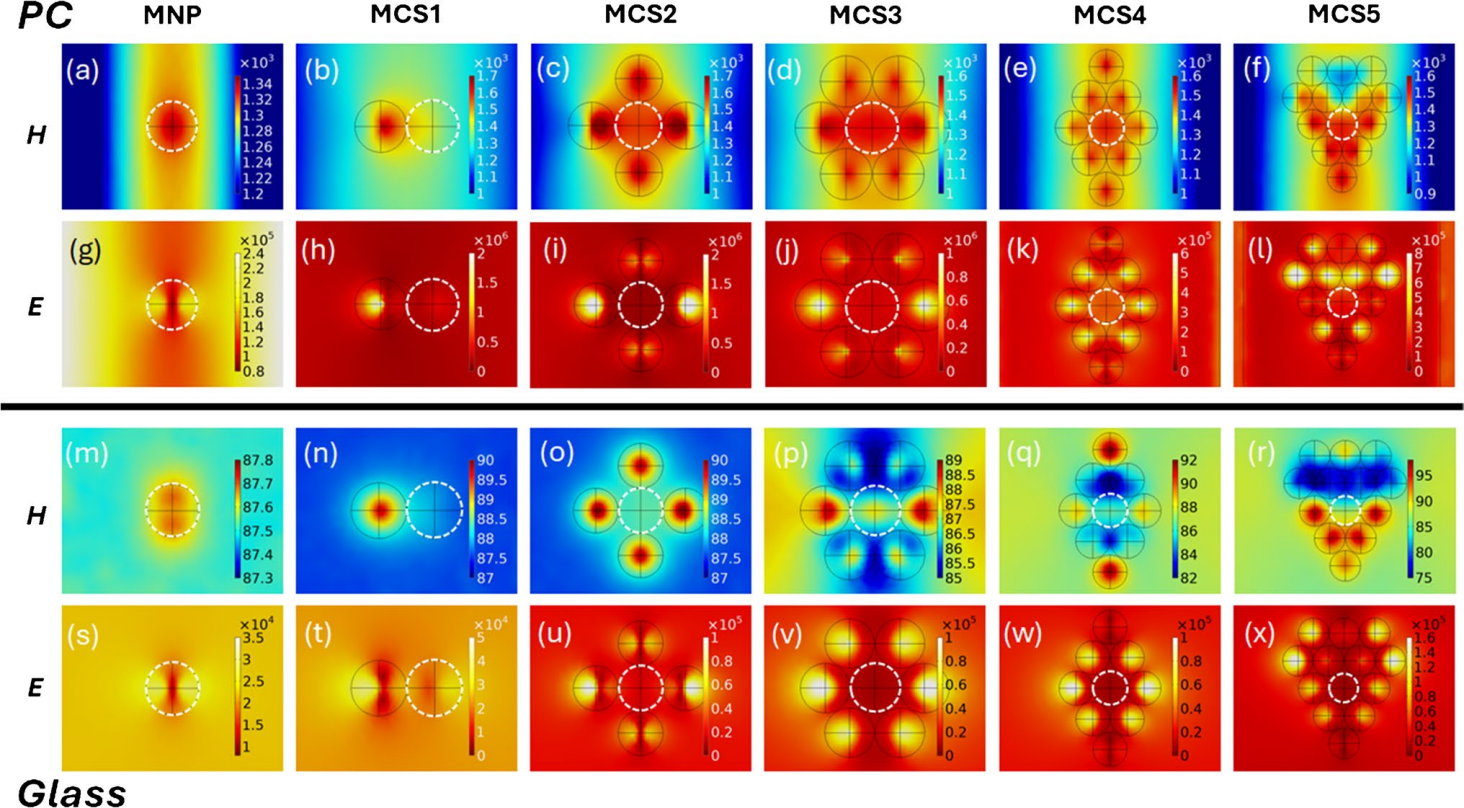
E-field and H-field distributions of plasmonic CSs and magneto-plasmonic CSs (MCSs). **a**–**f** H-field and **g**–**l** E-field intensity distribution of magnetic NP and MCSs 1–5, with the simulations performed over the PC substrate. **m**–**r** H-field and **s**–**x** E-field intensity distribution of magnetic NP and MCSs 1–5, with the simulations performed over the glass substrate. The dotted white line indicates magnetic Fe_3_O_4_ NP and the continuous black line represents plasmonic AuNP

While earlier reports establish the generation of E-field and H-field local enhancement with engineered configurations, interfacing magneto-plasmonic nano-assemblies near optically active substrates such as PCs is of particular interest as the substrate can further boost the local field intensities through constructive interference and energy funnelling into the existing hottest hotspots [53, 96]. In light of these observations, we performed COMSOL Multiphysics simulations to explore the interplay between NP composition and substrate effects on local field enhancement. As presented in Fig. 4, we modelled the E-field and H-field distributions for both pristine plasmonic gold CSs (based on Au NPs only) and magneto-plasmonic assemblies (Au + Fe₃O₄ NPs, written as MCSs) under transverse magnetic (TM) polarization. In this figure, the continuous black line drawn structures are AuNPs and dotted white line structures are Fe₃O₄ NPs, with the multi-particle arrangement of NPs in the nano-assembly considered as a representative case (as the structural arrangement in the CSs and MCS are anisotropic as seen in TEM images). Simulations were carried out for two substrate conditions: a standard glass substrate and a 1D PC grating engineered to support GMR at the excitation wavelength.

The results from simulations demonstrate a marked enhancement of both E-field and H-field intensities in the MCSs as compared to pristine plasmonic systems (CSs). In spite of its less plasmonic activity, the inclusion of Fe₃O₄ introduces additional pathways for field concentration via magnetic dipole resonances and field confinement at the NP junctions [97]. This is particularly prominent in the inter-particle gaps and at the periphery of the hybrid assemblies, where co-localized E- and H-hotspots emerge [57, 70, 98]. Moreover, we observed a significant amplification of both field components when the nano-assemblies are placed on the PC substrate rather than glass. We also noted that while E-field enhancement was around an order of magnitude on PC (vis-à-vis glass), the H-field enhancement was around two orders of magnitude higher. This is because the PC acts as a resonant optical cavity, enhancing the interaction between the incident light and the NPs that constitute the nano-assembly. This substrate-induced resonance leads to localized hybrid modes, which are a combination of the GMR of the PC and the LSPR of the NPs, hence culminating in synergistic E- and H-field hotspots with a magnitude of peak intensities higher than on glass.

**Fig. 5:**
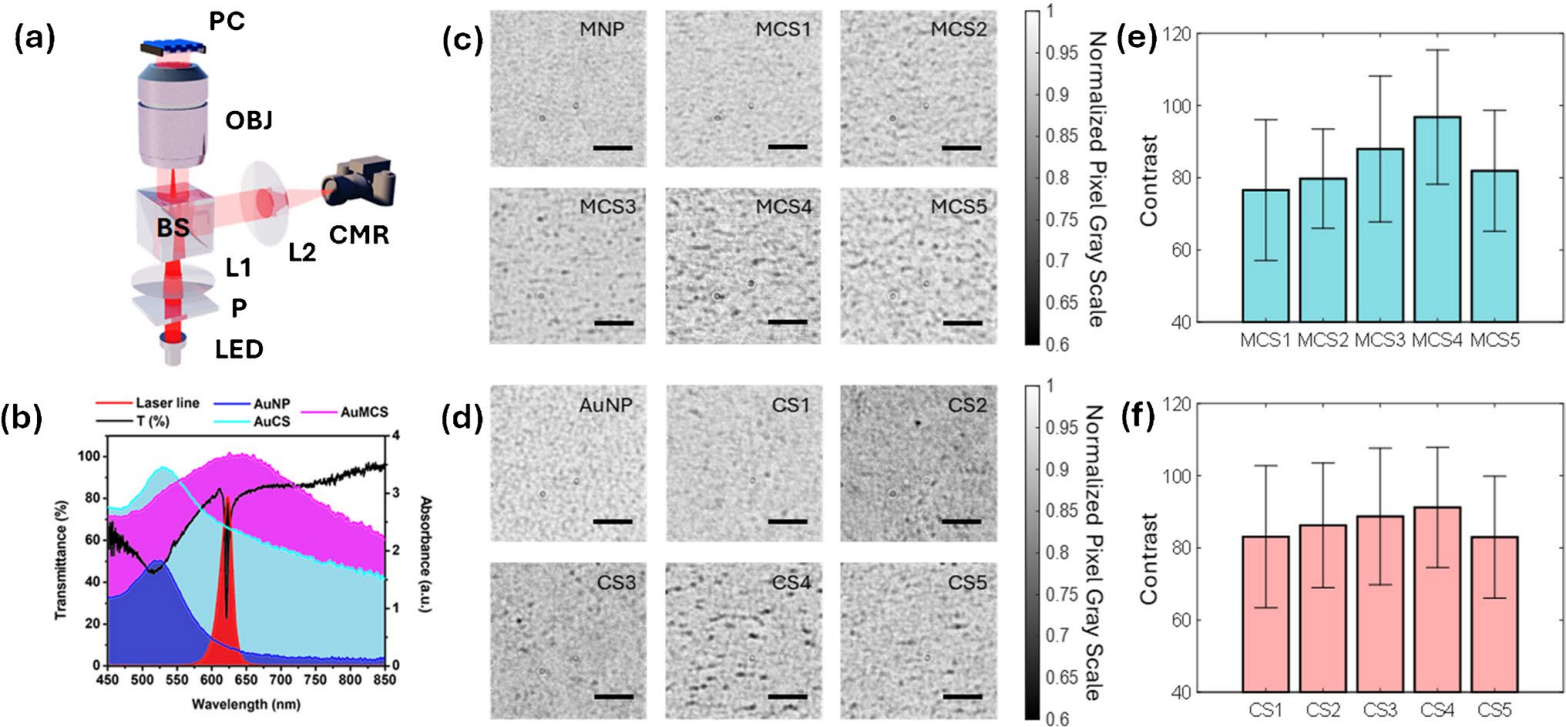
Photonic crystal absorption microscopy, optical coupling, and contrast analysis. **a** PRAM optical setup comprising LED, polarizer (P), lenses (L1, L2), beam splitter, objective, and camera. **b** Overlap of absorbance spectra of AuNP, AuCS, and AuMCS with the transmittance data of the PC and laser line used. PRAM images of **c** MCSs [1-5] and **d** CSs [1-5]. The scale bar in **c** and **d** is 20 µm. PRAM contrast analysis of **e** MCSs [1-5] and **f** CSs [1-5]

These observations reinforce the emerging paradigm of PC substrate-driven hybrid E- and H-field hotspots, where material composition and optical confinement may be co-engineered to manipulate optical effects at the nanodimensions. The simultaneous enhancement of E- and H-fields within the same nanoscale region is hence expected to yield intriguing results when observed under the PRAM optical setup, with results presented in Fig. 5.

The conceptual schematic of the optical setup used in the PRAM measurements is shown in Fig. 5a, with details presented in the “Experiment and simulation” section. The absorbance spectra of AuNPs, AuCS, and AuMCS are overlapped with the transmittance dip of the PC and the laser line spectra, all obtained experimentally. The AuCS and AuMCS are chosen for sample variants CS4 and MCS4 from Figs. 2c and 3c, respectively. We have chosen these for representation here because these variants yielded the highest contrast in each of their categories (discussed in the following paragraphs). From Fig. 5b, we note that there is an excellent overlap between the transmittance spectra of the PC resonance and the laser line under TM polarization. Furthermore, the absorption coefficient of AuNPs peaks at ~ 520 nm and decreases for higher wavelengths, showing almost negligible absorption at the resonance of the PC/laser line. Further, while the AuCS4 presents an increase in the absorption coefficient at higher wavelengths close to PC resonance, a red-shift is observed for AuMCS4, showing an excellent overlap of its plasmon resonance with that of the PC resonance. It is important to note that combining plasmonic and magnetic nanostructures has led to the emergence of novel physical phenomena such as magneto-plasmon-enhanced Faraday rotation, magnetic-field-induced Fano resonances, magnetic SPPs, and artificial magnetic metamaterials exhibiting negative permeability [65, 72, 88]. Although applications have rapidly expanded into multifunctional biosensors, magneto-optical modulators, light-controlled actuators, and theranostic platforms, the optical contrast response of magneto-plasmonic cryosorets on a PC interface is not reported.

We calculated the signal intensity through a two-step computation of the average contrast of each dark spot generated by the bound NPs or cryosorets. First, the signal spots were recognized with our PRAM image processing algorithm [1, 3, 5, 26]. The algorithm utilized area, pixel intensity, roundness, and Euler number of each spot as factors to screen the signal from non-nanoparticle features in accordance with our earlier works [5]. Due to the spectral misalignment of the LSPR of AuNP, MNP, and the PC’s GMR, there is not a dark spot whose pixel intensity exceeds the algorithm threshold in the 20-nm MNP and AuNP images; hence, no NPs are recognized, as shown in Fig. 5c and d. Second, the contrast was calculated through subtracting the maximum intensity in the image (mean value of the brightest 1000 pixels) from the mean intensity of pixels within the nanoparticle spot (consists of hundreds of pixels). The result is shown in Fig. 5e, f. Figure 5c shows representative cropped PRAM images (FOV: 80 × 80 μm^2^) for Fe_3_O_4_-involved MCSs. Through the calculation, we observed a growing contrast of MCS1-4 as the number of NPs per assembly increased; however, the contrast dropped when more NPs were further aggregated, as shown in Fig. 5e. We attributed this phenomenon to the less dwindled coupling effects between the oversized MCS5 as compared to MCS4, as particle sizes much higher than an optimum limit would render unfavorable optical effects with the evanescent field coupling. Additionally, the nano-assembly of more NPs demonstrates a larger absorption cross-section, thereby increasing the likelihood of overlapping between adjacent nano-assemblies, and hence resulting in fewer particles being exposed to the illumination and decreased effective absorption. The same phenomenon was also observed in the cryosorets consisting of AuNPs only, as shown in Fig. 5d and f. At this juncture, it is worth noting that the trend observed here, with an initial increase and further decrease in the signal contrast, is in line with our speculations from earlier experimentation of nano-assemblies in interferometric scattering microscopy [55], surface plasmo-coupled emission (SPCE) [53], and photonic crystal-coupled emission (PCCE) experimental measurements [62, 76].

Visualizing the photo-plasmonic effects at the surface of PC following the laser beam presents intriguing inferences. Firstly, the laser beam is incident on the PC surface via a polarizer (Fig. 5a). As the PC is engineered to generate a standing wave with high surface field intensity at this wavelength of laser line and polarization (TM), the PC is set to show GMR with a sharp dip in the transmittance (Fig. 5b) upon illumination. When the nano-assemblies are juxtaposed on the PC under such resonant conditions, the coupling occurs between the GMR of PC and the LSPR of the nanomaterial. The nano-assembly with optimum number of NPs per assembly renders the highest contrast in PRAM. The physical basis for enhanced contrast observed for magneto-plasmonic cryosorets vis-à-vis pristine plasmonic cryosorets is based on the interplay between E- and H-field plasmonic modes discussed in Fig. 4. While AuNPs exhibit strong electric dipolar resonances in the visible regime, Fe₃O₄ contributes magnetic responses that are weak in isolation but can be enhanced via near-field coupling with the plasmonic component. This hybrid configuration allows co-localization of EM hotspots and magnetically active regions, effectively merging optical field enhancement, magnetic effects, and uniform field distribution across the three-dimensional nano-gaps of cryosorets.

Unlike conventional molecular absorbers, where extinction arises solely from absorption, nanoparticle-based systems exhibit dual contributions, namely, absorption and scattering, and both of which depend on material and size [99, 100]. In the case of plasmonic NPs, size-dependent plasmonic oscillations can significantly amplify the scattering component [62, 76, 101].

**Fig. 6:**
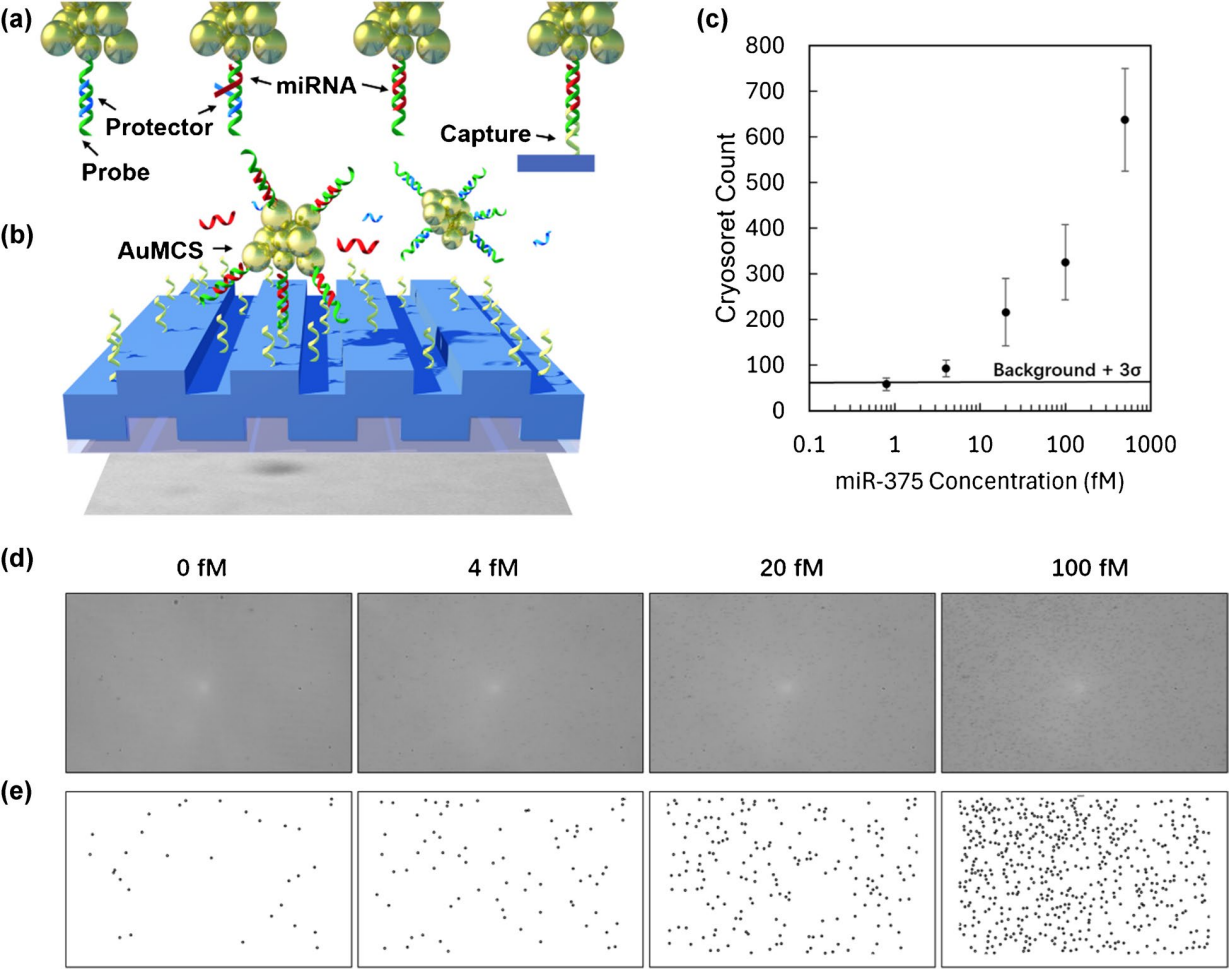
Assay demonstration of magneto-plasmonic CSs for detection of microRNA-375-3p using PRAM. **a** AC + DC assay using toehold-mediated strand displacement with DNA-functionalized AuMCS. **b** Capture on PC allows digital counting using PRAM. **c** Detection of miR-375 using AuMCS on PRAM, with five-fold serial dilutions tested (*n* = 3). The background is the average of three trials with no miR target plus the standard deviation (black line). **d** Raw PRAM images of four tested concentrations. **e** PRAM-cryosoret images that are processed and counted, with representative dark circles marking particles

To demonstrate the utility of nanoparticle assemblies on PRAM, DNA-functionalized magneto-plasmonic cryosorets were used in an Activate Capture + Digital Counting assay for miR-375-3p detection. We have used a methodology that has been established and presented with complete details in our earlier works [26]. The AC + DC assay (summarized in Fig. 6a, b) uses toehold-mediated strand displacement reactions to trigger the release of a protector strand when the complementary target sequence is present, allowing the “activated” probe-functionalized AuMCSs to bind to a capture sequence functionalized on the PC surface to be digitally counted. If no target is present, the protector prevents the AuMCSs from binding. This allows a single target microRNA to activate and capture a cryosoret for PRAM detection. The assay was run for six different miR-375 concentrations in five-fold dilutions spiked into 1xTE, 12.5 mM MgCl_2_ buffer. The images were collected after 30 min and counted using image processing, with the details of the sequences and steps involved in the assay presented in the experimental section. Although the limit of detection (LOD) achieved with the cryosoret nano-assemblies in this study (~ 2.5 fM) is not significantly lower than previously reported values (~ 160 aM) [26], the approach presented here offers a simpler and more cost-effective alternative, avoiding the need for expensive maleimide-functionalized kits traditionally used for DNA attachment. The reliable performance of the AuMCS in detecting target analytes highlights its potential for multiplexed detection, particularly due to the tunable absorbance spectra achievable with cryosoret nano-assemblies composed of multi-metallic or multifunctional nanomaterials.

## Future scope and perspectives

Furthermore, it is informative to present the future scope and perspectives of this research under PRAM imaging setup, especially with regard to the insights that can be drawn by noting the behavior of nano-assemblies on PC substrate. While the isolated NPs remained undetected due to the spectral mismatch between their localized surface plasmon resonance (LSPR) and the guided-mode resonance (GMR) of the PC, the formation of cryosoret nano-assemblies from dimers to higher-order multimers resulted in a pronounced PRAM signal. This contrast enhancement that is absent in single NPs underscores the emergence of inter-particle plasmonic coupling that effectively shifts and broadens the absorption profile of the nano-assemblies into the resonance window of the PC substrate. This observation marks a significant advance over our earlier works (using plasmonic and dielectric NPs of various shapes) and highlights a transformative concept: When a NP exhibits high specificity for a target biomolecule but lacks resonance alignment with the PC, we can circumvent the need for tuning the PC substrate by instead engineering tailored cryosoret nano-assemblies. These nano-assemblies inherently exhibit enhanced absorption at longer wavelengths (longitudinal plasmons), thus bridging the spectral gap and enabling detection without substrate modification. This modular approach can significantly streamline the multiplexing of various analytes on a single chip, which is otherwise not possible when relying solely on monodisperse, resonance-matched pristine NPs.

Furthermore, the observed nonlinear relationship between the contrast signal and the number of NPs per nano-assembly suggests a complex interplay between EM field localization and scattering losses. Such behavior opens intriguing opportunities for the rational design of NP assemblies that balance field enhancement and absorption cross-section to maximize imaging contrast, the concepts of which have been demonstrated using interferometric scattering effects in our recent report [55]. Future work could involve optimization of assembly geometries (e.g., trimers, tetramers, fractals), material properties (such as metal-dielectric alloys, core–shell, decorated hybrids), and shapes (nanorod, nanourchin, nanostar, nanocubes) to exploit near-field coupling and photo-plasmonic confinement effects. Currently, the ongoing experiments aim to understand the spectral shifts in terms of peak wavelength and intensity by systematically varying the nanoparticle composition (e.g., Au, Ag, Pt, Pd, Al) and employing hybrid and dielectric materials (e.g., TiO₂, SiO₂). Such shifts, which are governed by modulation of real and imaginary components of the composite dielectric function of the NP-environment system at the resonance wavelength of PC, are expected to offer new routes for finely tuned biosensor responses, especially for label-free detection strategies.

Hence, an exciting frontier lies in the incorporation of magnetic nanomaterials within these plasmonic assemblies to realize magneto-plasmonic cryosorets in three directions: (i) using pristine magnetic NP assemblies, (ii) using hybrid plasmonic + magnetic nano-assemblies, (iii) using dielectric nanoparticles that are doped with certain species to generate magnetic effects. Such hybrid constructs can enable remote actuation via external magnetic fields, facilitate accelerated bioassay workflows, and allow dynamic manipulation or spatial patterning of sensing elements, which are important features particularly valuable in point-of-care diagnostics. Moreover, the Janus nature of these multifunctional systems could also be harnessed for combined optical and magnetic readouts, unlocking synergistic sensing modalities and novel mechanochemical transduction mechanisms.

## Conclusions

The crysoret nano-assemblies of pristine plasmonic AuNPs and their hybrids with magnetic Fe_3_O_4_ NPs are synthesized and interfaced on the PC interface and investigated using photonic resonator absorption microscopy (PRAM) as well as using COMSOL Multiphysics simulations. The hybrid coupling between the guided-mode resonance of the underlying PC under TM polarization presented enhanced contrast of the nano-assemblies while their individual counterparts could not be detected. The magneto-plasmonic coupling effects in hybrid cryosorets of plasmonic Au and magnetic Fe_3_O_4_ NPs presented better contrast as compared to that observed with pristine plasmonic Au-based cryosorets. The simulations presented insights pertaining to such experimental observations where the electric and magnetic flux densities are enhanced for hybrid cryosorets due to the hybrid coupling in magneto-plasmonic nano-assembly. While the isolated metal NPs present electric field hotspots, the emergence of E-field and H-field hotspots in magneto-plasmonic cryosorets over glass interface is established. Additionally, such hotspots are tremendously augmented when the nano-assemblies are interfaced on the PC interface due to the hybridization of delocalized Bragg and localized Mie plasmons of cryosorets with the GMR of the underlying PC substrate.

In summary, the work reported here lays a foundational framework for the use of nano-assemblies on PCs as tunable, high-contrast probes in label-free sensing. Unlike traditional core–shell or layer-by-layer constructs, our assemblies preserve the native surfaces of both NP types. This cryosoret-mediated self-assembly method not only avoids the need for surface linkers, but also ensures that both the magnetic and plasmonic interfaces remain accessible for external functionalization. Such a feature is crucial for biosensing, where nanoparticle surface chemistry dictates specificity and sensitivity. The concept of nano-assembly-mediated resonance bridging introduces a flexible paradigm that could be extended across a variety of PC configurations, including 1D gratings, 2D slab resonators, and 3D colloidal crystal-based PCs (opals and inverse opals), each offering unique opportunities for enhanced light-matter interactions. Coupling these structures with machine-learning-based design of NP configurations and real-time spectral feedback could lead to adaptive, intelligent biosensors tailored for personalized diagnostics and dynamic opto-fluidic environments. We envisage the development of different types of bioassays (such as sandwich type for example) where the PC substrate is engineered with capture molecules, followed by selective attachment of nano-assembly tagged secondary antibodies, where only in the presence of an analyte the sandwich would manifest, thus enabling highly specific and sensitive multiplexing. Moreover, the simultaneous realization of E-field and H-field intensity distributions in magneto-plasmonic hybrids holds promise for the next-gen integrated biosensors, magneto-optical modulators, and linear-nonlinear hybrid optical devices.

## Data Availability

The data that support the findings of this study are available from the corresponding authors upon reasonable request.
